# Gut Microbiome Recovery in *Clostridioides difficile* Infection Patients Receiving Multi-Strain Probiotics During Convalescence: A Prospective Pilot Series of Longitudinal Dynamics

**DOI:** 10.3390/diseases14020077

**Published:** 2026-02-18

**Authors:** Dorin Novacescu, Talida Georgiana Cut, Adelina Baloi, Alexandra Herlo, Ioana-Melinda Luput-Andrica, Andra Elena Saizu, Amelia Uzum, Maria Daniela Mot, Flavia Zara, Dorel Sandesc, Voichita Elena Lazureanu, Adelina Marinescu

**Affiliations:** 1 Department II of Microscopic Morphology, Victor Babes University of Medicine and Pharmacy Timisoara, E. Murgu Square, No. 2, 300041 Timisoara, Romania; novacescu.dorin@umft.ro (D.N.); flavia.zara@umft.ro (F.Z.); 2 Department XIII, Discipline of Infectious Diseases, Victor Babes University of Medicine and Pharmacy Timisoara, E. Murgu Square, No. 2, 300041 Timisoara, Romania; alexandra.mocanu@umft.ro (A.H.); ioana.luput-andrica@umft.ro (I.-M.L.-A.); andra.saizu@umft.ro (A.E.S.); lazureanu.voichita@umft.ro (V.E.L.); adelina.marinescu@umft.ro (A.M.); 3 Department X—Surgery II, Discipline Anesthesia and Intensive Care, Victor Babes University of Medicine and Pharmacy Timisoara, E. Murgu Square, Nr. 2, 300041 Timisoara, Romania; adelina.baloi@umft.ro (A.B.); sandesc.dorel@umft.ro (D.S.); 4 Doctoral School, Victor Babes University of Medicine and Pharmacy Timisoara, E. Murgu Square, No. 2, 300041 Timisoara, Romania; 5 Multidisciplinary Doctoral School, Vasile Goldis Western University of Arad, 310025 Arad, Romania; 6 Department of General Medicine, “Vasile Goldis” Western University of Arad, Blvd. Revolutiei, No. 96, 310025 Arad, Romania; mot.dana@uvvg.ro

**Keywords:** *Clostridioides difficile* infection, intestinal microbiome, post-infectious dysbiosis, healthcare-associated infections in Romania, gut microbiome surveillance and recovery, gut microbial diversity, *Proteobacteria* expansion, 16S rRNA gene sequencing, Omni-Biotic 10, multi-strain probiotic

## Abstract

**Background/Objectives:** *Clostridioides difficile* infection (CDI) is a major healthcare-associated infection associated with profound antibiotic-induced gut microbiome disruption that frequently persists after clinical resolution. This pilot study aimed to characterize early post-infectious gut microbiome recovery following an inaugural CDI episode and to descriptively assess microbiome remodeling during adjunctive multi-strain probiotic supplementation. **Methods:** Adult patients with mild-to-moderate CDI were prospectively enrolled after completing standard antimicrobial therapy and received a 30-day course of a high-potency, 10-strain probiotic formulation. Stool samples were collected before and after supplementation and analyzed using 16S rRNA gene sequencing with microbiome-inferred functional profiling, alongside targeted screening for enteric protozoa and yeasts. **Results:** Five patients completed paired analyses. At baseline, all patients exhibited severe dysbiosis characterized by markedly reduced microbial diversity, depletion of *Actinobacteria* and short-chain fatty acid-producing taxa, expansion of *Proteobacteria*, and unfavorable inferred metabolic signatures. After supplementation, four of five patients were observed to exhibit increased microbial diversity and partial improvement in global dysbiosis indices. Microbiome recovery was heterogeneous and non-linear, involving variable reductions in *Proteobacteria*, recovery of *Actinobacteria*, or both, with incomplete normalization of taxonomic balances and inferred functions. Enterotype shifts were observed in three patients, consistent with ecological reorganization rather than full restoration. Baseline protozoal colonization resolved in affected patients, while fungal dynamics showed clearance or species-level replacement. No early CDI recurrences were observed during follow-up. **Conclusions:** Interpretation is limited by the single-arm design without a control group, which precludes distinguishing supplementation-associated changes from natural post-antibiotic recovery. Even so, our findings highlight the complexity and inter-individual variability of early post-CDI microbiome recovery and support further investigation of integrative microbiome profiling to describe post-infectious dysbiosis dynamics.

## 1. Introduction

*Clostridioides* (*C.*) *difficile* (formerly *Clostridium difficile*) infection (CDI) is a leading cause of healthcare-associated diarrhea and colitis, particularly in hospitalized and elderly patients [1,2]. This anaerobic, spore-forming bacterium produces potent toxins that damage the colonic mucosa, leading to manifestations ranging from mild diarrhea to life-threatening pseudomembranous colitis, ileus and colonic perforation [3,4]. Consequently, CDI imposes a major clinical and economic burden due to its high recurrence rate—~20–35% of patients experience at least one relapse after the initial episode [5]. Recurrent CDI can initiate a vicious cycle of illness, where each subsequent antibiotic treatment further perturbs the gut microbiome and predisposes to yet another recurrence [6].

Gut microbiome plays a crucial role in human health by aiding digestion, modulating immunity, and preventing pathogen overgrowth [7,8]. Disruption of the normal gut microbiome (“dysbiosis”) is a central factor in CDI pathogenesis and recurrence. Antibiotic exposure—the main predisposing factor for CDI—depletes the diversity and balance of the intestinal microbiome, weakening colonization resistance against *C. difficile* [9]. Healthy gut microbial communities normally prevent pathogen overgrowth by competing for nutrients, producing inhibitory metabolites such as short-chain fatty acids (SCFAs) and secondary bile acids, and maintaining mucosal integrity [10]. When this protective flora is eroded, *C. difficile* spores can germinate and proliferate unchecked, as the natural colonization barriers, i.e., the checks and balances on pathogen proliferation, are lost [11]. CDI-related dysbiosis typically involves depletion of beneficial commensals (e.g., *Bifidobacterium* and butyrate-producing *Firmicutes* such as *Lachnospiraceae* and *Ruminococcaceae*) and expansion of opportunistic, pro-inflammatory taxa, such as *Proteobacteria* [12]. The loss of butyrate-producing bacteria is particularly detrimental, as butyrate, an SCFA-like mediator, supports gut epithelial integrity, modulates immune responses, and directly inhibits *C. difficile* growth [13].

Notably, antibiotic-induced microbiota disruption not only triggers initial CDI, but also inherently further increases susceptibility to recurrent infection after initial treatment [14,15]. Indeed, studies have shown that standard CDI therapies, such as oral vancomycin, can cause drastic alterations in the gut microbiome—eliminating beneficial organisms (e.g., *Bacteroidetes*) and allowing blooms of pathogens—with persistence of dysbiosis long after initial treatment in some patients [16,17]. Thereafter, this incomplete microbiota recovery provides a fertile ground for *C. difficile* spores to proliferate once more and cause recurrence, thus emphasizing the need for post-treatment microbiome rehabilitation in CDI.

Given the aforementioned pivotal role of gut microbiome disruption in CDI pathogenesis, there is implicitly strong rationale to focus on gut flora restoration as a therapeutic objective, to be included as standard practice within the clinical management of CDIs, as a means of re-establishing colonization resistance and reducing recurrence. Fecal microbiota transplantation (FMT) offers the most direct and consistent evidence for this gut microbiome rehabilitation approach, through its established and well-documented success in treating recurrent CDIs, i.e., achieving curability rates of ~80–90%, as reported by multiple studies [18,19]. By reintroducing a diverse, protective gut microbial population from a healthy donor, FMT has repeatedly demonstrated the ability to break the cycle of dysbiosis and CDI relapse where antibiotics alone have often failed, underscoring the importance of microbiome diversity and resilience in CDI prophylaxis. However, FMT is not widely available, constituting an invasive, costly and logistically complex procedure, thereby motivating an amounting interest in safer, standardized, alternative microbiome-based interventions [20]. In this context, probiotics—defined as live microorganisms that confer a health benefit when administered in adequate amounts—have emerged as a potentially relevant adjunct tool for normalizing the post-CDI gut microbiome [21,22].

Probiotic therapy in CDI has been proposed as an adjunctive strategy to support post-antibiotic microbiome recovery by introducing beneficial microbial taxa that may contribute to colonization resistance against *C. difficile*, modulation of local metabolic activity, and maintenance of mucosal barrier integrity and defenses. Amounting studies have investigated probiotics for preventing antibiotic-associated diarrhea (AAD) and CDI, with mixed, yet generally encouraging, findings overall. Heretofore, although some meta-analyses have already suggested that prophylactic probiotics may reduce CDI incidence in antibiotic-treated patients [23,24], further randomized controlled clinical trials have yielded inconsistent results and thus far been unable to unequivocally validate this notion [25]. Clinically, various probiotic species have been tested, including *Lactobacillus* and *Bifidobacterium* strains [26], as well as yeasts such as *Saccharomyces boulardii* [27], with all having shown, albeit somewhat inconsistently, potential benefits in reducing *C. difficile* incidence and/or recurrence in select populations. Notably, *S. boulardii* used alongside standard antibiotic therapy has been reported to reduce CDI recurrence, likely by inhibiting pathogen overgrowth, while also facilitating proteolysis of *C. difficile* toxins [28,29,30].

The current heterogeneity in probiotic strains investigated, dosage and duration of probiotic treatments, and overall study designs has led to proportional variability in reported outcomes. Accordingly, current guidelines remain cautious about recommending routine probiotic use in CDI, due to insufficient high-quality evidence supporting a clear benefit [31]. Nevertheless, this concept of using probiotics to counteract post-antibiotic gut dysbiosis remains biologically plausible and overall data trends clearly support further investigation—particularly of multi-strain formulations and their mechanistic impact—to hopefully clarify the optimal approach and/or expected benefits in CDI specifically [32,33].

In fact, multi-strain probiotic formulations have already been proposed as a promising avenue to improve clinical outcomes in CDI [34,35]. Since CDI perturbs many facets of gut microbiome composition and host physiology, it seems intuitively accurate that no single probiotic strain may suffice to restore balance. Instead, a consortium of different, complementary, beneficial microbial lineages might be better equipped to more effectively restore biodiversity, by providing additional synergistic effects—enhanced colonization resistance, mucosal repair support, and inflammation suppression [27,32,35,36]. In corroboration of this rationale, over a decade ago, an early multi-strain probiotic formulation (Ecologic^®^ AAD/Omni-Biotic^®^ 10) achieved complete clinical resolution in a small cohort of ten CDI patients at high-risk of recurrence, i.e., no relapse with adjunct probiotics [37], highlighting the potential synergistic benefits of a broad-spectrum multi-strain approach.

Omni-Biotic^®^ 10 (OB10), a formulation containing ten well-characterized probiotic strains [38], designed to replenish beneficial gut bacteria after antibiotic exposure, has since shown reliable activity regarding gut microbiome diversity and suppressing Gram-negative pathogens in both clinical and experimental settings [39]. However, rigorous evidence regarding the impact of multi-strain probiotics on host gut microbiome in CDI remains limited. Most prior studies have focused on clinical endpoints (e.g., diarrhea or recurrence rates) rather than detailed microbiome recovery profiles in CDI patients. In particular, there is a knowledge gap regarding how such interventions affect the composition and metabolic activity of the gut microbiome during the post-CDI convalescence period. Even so, understanding how probiotic therapy reshapes gut microbial ecosystems could potentially inform prophylaxis strategies and improve long-term outcomes in CDIs.

To address this knowledge gap, we conducted a pilot study to characterize gut microbiome recovery following initial CDI treatment and to descriptively assess microbiome transformations during a 30-day course of adjunct multi-strain probiotic (OB10) supplementation. Thus, we examined changes in intestinal microbiota diversity, key microbial populations, and inferred metabolic profiles in patients recently treated fully for an inaugural CDI episode, before and after a full month of daily probiotic administration. We focus on key microbiome parameters—including overall diversity, the *Firmicutes*/*Bacteroidetes* (F/B) ratio (often used as a general index of microbiota balance), the *Actinobacteria*/*Proteobacteria* (A/P) ratio, levels of beneficial genera vs. opportunistic bacteria, and the presence of pathogenic microbes or inflammation-related taxa—to observe any fluctuations associated with the probiotic period. By comparing microbiome profiles before vs. after supplementation, we aimed to describe whether microbiome remodeling toward a more eubiotic profile would be observed during early post-CDI convalescence in the context of adjunct multi-strain probiotic supplementation. This pilot analysis aims to describe the microbiome-level changes during early post-CDI recovery with adjunct multi-strain probiotic therapy and provide further evidence to inform microbiota-targeted strategies for reducing CDI recurrence.

## 2. Materials and Methods

### 2.1. Study Design and Participants

**Figure 1:**
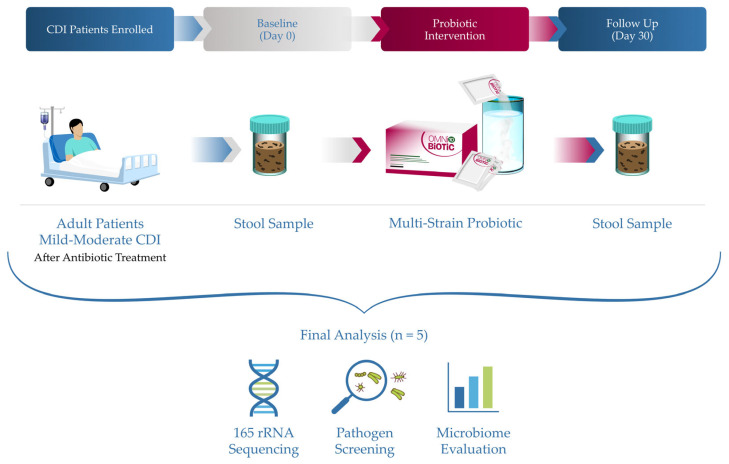
Study design and longitudinal sampling framework of the post-Clostridioides difficile infection (CDI) probiotic pilot study. Adult patients recovering from a first, mild-to-moderate CDI episode were prospectively enrolled after completion of standard antimicrobial therapy. Stool samples were collected at baseline (Day 0) and after a 30-day course of adjunctive multi-strain probiotic supplementation (Day 30). Paired stool samples were analyzed using 16S rRNA gene sequencing with microbiome-inferred functional profiling and targeted screening for enteric protozoa and fungi. Five patients completed paired longitudinal analyses. N.B.: created by our graphic design expert Marius Filip.

This pilot study was conducted as a single-center, prospective observational study involving adult patients recovering from a first (inaugural, non-recurrent) episode CDI, following completion of standard antibiotic therapy, within the Infectious Diseases II ward of the “Victor Babes” Clinical Hospital Timisoara. The primary objective was to characterize post-antibiotic intestinal dysbiosis after clinical CDI resolution and to describe longitudinal microbiome changes observed during a 30-day period of adjunctive probiotic supplementation, rather than to establish efficacy. The overall study design, sampling timeline, and analytical workflow are summarized in Figure 1.

As a pilot initiative, secondary objectives included feasibility metrics (retention, adherence, and successful paired sampling) to inform the design of a future controlled trial. Inclusion criteria required age ≥18 years, no prior history of CDI and/or no ongoing recurrent infection, no other acute infections or illnesses, and willingness to follow the intervention protocol and provide stool samples at designated time points.

Initially, we analyzed data from seven adult patients (4 males, 3 females; age range 27–79), all with laboratory-confirmed CDI of mild-to-moderate severity, with each having completed a standard course of antibiotic therapy, according to contemporary guidelines, and achieving clinical resolution of CDI just prior to enrollment. None had received fecal transplant. Key clinical metadata (age, sex, comorbidities, context of on-set, treatment course) were recorded; notably, most patients were older adults with coexisting conditions, commonly associated with CDI (e.g., recent hospitalization and/or antibiotic use). The most common comorbidities were cardiovascular disease and metabolic conditions (i.e., diabetes mellitus), but no patient was immunosuppressed.

Each patient’s baseline stool sample was collected at the end of CDI antibiotic therapy (defined as Day 0, pre-intervention), and a follow-up sample was collected ~30 days after the probiotic intervention (Day 30, post-intervention). Two patients were lost to follow-up prior to the Day 30 evaluation and were excluded from outcome analyses. Consequently, the final analysis included five patients with paired baseline and post-intervention microbiome data.

All patients provided informed consent for stool analysis. The study analyzed de-identified data; it did not involve any intervention beyond standard care and adjunct probiotic. All procedures were carried out in accordance with the Declaration of Helsinki and applicable national guidelines. Even so, this initiative was formally approved by the Ethics Committee of the “Victor Babes” Hospital of Infectious Diseases and Pneumophtisiology Timisoara (protocol code 6631/13 July 2021, renewed 3082/4 April 2023).

### 2.2. Intervention Protocol

All participants received general dietary and lifestyle recommendations, intended to support gut health during post-CDI recovery, but not formally controlled and/or monitored as an interventional variable. Patients were advised to consume a balanced, high-fiber, plant-rich diet with diverse vegetables, fruits, and whole grains, while limiting processed foods, animal fat, and added sugars. A target of at least 25–30 g/day of dietary fiber was set to ensure ample substrate for colonic fermentation. We acknowledge that the high-fiber dietary recommendations constitute a major, uncontrolled, confounding variable; however, this mirrors real-world clinical advice given to post-CDI patients. Even so, we explicitly note that these structured diet modifications may be an important potential factor that could have influenced the microbiome outcomes independently.

Refined sugar intake was restricted (<5 g/day of free sugars), to discourage yeast and saccharolytic *Proteobacteria* overgrowth [40]. Wheat-based products were minimized due to the presence of amylase–trypsin inhibitors that can irritate the gut mucosa and exacerbate inflammation in sensitive individuals [41], whereas gluten-free whole grains (oats, brown rice, quinoa) and other resistant starch foods were recommended. Red meat intake was limited to ≤2 servings/week, especially important for those with high trimethylamine N-oxide (TMAO)-producing bacteria, in order to reduce dietary choline and carnitine substrates [42].

Additional recommendations included adequate hydration (approximately 2–3 L/day of water or unsweetened herbal teas), regular physical activity, and avoidance of smoking and excessive alcohol consumption. Use of unnecessary medications, known to disrupt gut microbiome composition (e.g., proton pump inhibitors, non-steroidal anti-inflammatory drugs, repeated antibiotic courses), was discouraged when clinically feasible. Additionally, patients were introduced to the general principle that any future antibiotic therapies should be accompanied by probiotic support. These diet and lifestyle measures provided a foundational environment conducive to microbiome recovery.

The probiotic was aimed at restoring gut flora diversity and function in the post-infection phase. Following completion of CDI antibiotic therapy, participants underwent a 30-day course of multi-strain probiotic (Omni-Biotic^®^ 10 AAD, Allergosan, Graz, Austria). OB10 is a high-concentration probiotic formulated for antibiotic-associated dysbiosis/AAD, containing ten specially selected human gut bacterial strains, with a minimum of 5 × 10^9^ colony forming units (CFUs) of each strain, totaling ~50 × 10^9^ CFUs per dose (5 g sachet). The full composition includes *Lactobacillus acidophilus* (strains W55 and W37), *Lactobacillus plantarum* (W1 and W62), *Lactobacillus paracasei* (W20), *Lactobacillus rhamnosus* (W71), *Lactobacillus salivarius* (W24), *Bifidobacterium lactis* (W51), *Bifidobacterium bifidum* (W23), and *Enterococcus faecium* (W54). Patients were instructed to take one sachet (5 g) of the probiotic daily, dissolved in water, for 30 consecutive days after finishing antibiotic treatment [43]. The probiotic was taken on an empty stomach (typically before breakfast), as per manufacturer recommendations. Adherence to the probiotic regimen was reinforced through weekly follow-up calls, and all five completing patients reported full compliance with the 30-day course.

### 2.3. Sample Collection and Handling

Stool samples were collected at two time points for each patient (see Figure 1): baseline (Day 0), defined in this study as the first bowel movement sample obtainable after completing the CDI antibiotic course (and prior to starting probiotic supplementation), and post-intervention (Day 30)—a sample obtained at the end of the 30-day probiotic course (within ±3 days of Day 30). Participants were provided with a stool collection kit and detailed instructions for safe sample handling. Each fresh stool sample (approximately 10–20 g, midstream, avoiding urine or water contamination) was self-collected by the patient in a sterile screw-cap container. Patients kept the specimen refrigerated (4 °C) immediately after collection and delivered it to the study site within 24 h. Upon receipt, ~5–10 g of stool was transferred into two aliquots: (1) a plain screw-cap fecal specimen cup for conventional analyses and (2) a DNA stabilization tube (OmicSnap^®^, Biovis Diagnostik MVZ GmbH, Limburg, Germany) for molecular analysis. The latter contains a nucleic acid preservation medium recommended by the laboratory to maintain microbial DNA integrity during transport [44]. Samples were kept refrigerated (4 °C) until shipment.

All specimens were shipped by overnight courier, within 24–48 h of collection, to an external laboratory, i.e., Biovis Diagnostik MVZ GmbH (Limburg, Germany), for microbiome analysis. Shipments included cold packs, though the stabilization buffer allows for safe ambient-temperature transport. Upon arrival at the laboratory, samples were registered and immediately processed for downstream analyses. Each sample was labeled with a unique study code and time point (baseline or 30-day) to ensure proper pair matching. The laboratory personnel analyzing the specimens were independent of the clinical team; however, given the nature of the study, they were not blinded to sample timing (the lab reports included the sampling dates).

### 2.4. Microbiome Sequencing and Ancillary Testing

All stool analyses were performed at the accredited Biovis Diagnostik MVZ laboratory (Limburg, Germany), which specializes in integrative gut microbiome diagnostics. The comprehensive intestinal microbiome analysis used a molecular-genetic platform based on 16S rRNA gene sequencing of bacterial DNA isolated from stool [45]. Genomic DNA was extracted from approximately 200 mg of stool using a bead-beating enhanced extraction protocol and a commercial stool DNA kit (e.g., QIAamp DNA Stool Mini Kit, Qiagen, Hilden, Germany), according to the manufacturer’s instructions for maximum yields. The DNA extracts were quality-checked for purity and concentration using spectrophotometric analysis, and then forwarded for 16S rRNA gene amplicon sequencing [45].

The hypervariable regions V3–V4 of the 16S rRNA gene were polymerase chain reaction (PCR)-amplified from each sample using broad-range bacterial primers, and then subjected to next-generation sequencing (NGS) on an Illumina MiSeq platform (Illumina Inc., San Diego, CA, USA), to generate 2 × 300 bp paired-end reads, following standard protocols for microbiota profiling. This NGS approach identifies bacterial taxa by matching their unique 16S gene sequences to reference genome databases, determining which bacteria are present in each sample and in what relative quantities [46]. Bioinformatics processing was conducted with a standard pipeline: raw reads were quality-filtered, trimmed, and clustered into operational taxonomic units (OTUs) at 97% similarity. Taxonomic assignment of OTUs was performed by alignment against curated databases from the NIH Human Microbiome Project and other 16S reference libraries (SILVA 16S rRNA database, release 138) [47,48]. Abundance tables were generated to quantify each taxon’s representation as a percentage of the total bacterial community. The result was a comprehensive profile of the intestinal microbiota composition for each sample, including relative abundances of bacteria from phylum down to genus (and species level where resolvable).

In total, the Biovis microbiome panel provided quantitative results for over 250 parameters [49], spanning taxonomic composition and microbial functional markers. In addition to high-throughput sequencing, microbiome-derived functional markers were inferred based on the relative abundance of bacterial taxa known to harbor specific metabolic pathways (e.g., ammonia-producing, β-glucuronidase-bearing, phenol-producing bacteria), rather than direct quantification of stool metabolite concentrations. The laboratory also performed multiplex real-time PCR and culture-based methods to detect common enteric pathogens not easily captured by 16S sequencing. This included screening for *C. difficile* toxin genes, pathogenic *Escherichia coli* strains, and other enteric pathobionts, as well as yeast and parasite detection. Fungal culture and PCR were used to check for overgrowth of yeasts (including *Candida* spp.), and a multiplex PCR panel targeted protozoan parasites.

Conversely, the laboratory did not provide run-level metadata (e.g., per-sample read depth distributions, exact filtering thresholds, chimera detection method, or software version identifiers); therefore, fully independent replication of the computational pipeline is constrained. In addition, negative extraction blanks and mock community controls were not available for the present sample set in the laboratory report, and contamination assessment in the lowest-diversity baseline samples could not be formally performed. Microbiome-derived “functional” readouts in the Biovis panel represent inferred functional potential based on taxa associated with particular metabolic pathways and do not constitute direct stool metabolite quantification.

### 2.5. Key Microbiome Parameters

The following key microbiome parameters were evaluated for each sample, in the context of their clinical or functional significance, as detailed in Table 1 below:

Importantly, all microbiome indices used (Dysbiosis Index, Biodiversity Index, F/B ratio, A/P ratio, enterotype classification) are semi-quantitative and context-specific indicators. They provide a relative measure of microbiome status within this study’s framework and are not generalizable beyond this context.

### 2.6. Data Processing and Interpretation

Data analysis was primarily descriptive, given the pilot nature of the study and the small sample size (*n* = 5 paired samples). We tabulated changes in diversity indices, dysbiosis scores, and the direction of change (↑/↓/N, compared to healthy population benchmarks) for major taxa and functional markers. The F/B ratio was assessed qualitatively: patients with low *Bacteroidetes* and relatively higher *Firmicutes* were interpreted as having an elevated F/B ratio, whereas depletion of *Firmicutes* or dominance of *Bacteroidetes* indicated a low ratio. Similarly, the A/P ratio served as a proxy for mucosal dysbiotic stress, with baseline profile of “*Actinobacteria* ↓, *Proteobacteria* ↑” indicating a critically low A/P ratio (deficit of beneficial bifidobacteria alongside proteobacterial overgrowth). We tracked longitudinal within-subject changes during the supplementation period based on directional shifts and partial normalization patterns, without assigning binary responder/non-responder status.

To evaluate global microbiome restructuring beyond isolated metrics, principal component analysis (PCA) was performed using Python (version 3.10), with the scikit-learn and matplotlib libraries. Data were standardized (z-score normalization) prior to dimensionality reduction to account for scale differences between diversity indices and taxonomic relative abundances. Principal components were derived from the covariance matrix of these standardized variables (Biodiversity Index, Dysbiosis Index, and relative abundances of *Firmicutes* and *Proteobacteria*) to ensure equal weighting, with variance explained calculated from the eigenvalues. This multivariate approach allowed for the visualization of patient-specific trajectories from dysbiosis toward a eubiotic state. To ensure robustness given the limited sample size, PCA was restricted to these four orthogonal variables capturing ecological, clinical, and structural dimensions of the microbiome; additional taxonomic variables were excluded to avoid redundancy and minimize overfitting and collinearity given the small sample size (*n* = 5 paired samples). Given the small sample size (10 observations), PCA is presented herein as a descriptive visualization of within-subject displacement rather than a robust inferential model; component structure and apparent clustering are not expected to be stable or generalizable.

Finally, we assessed clinical microbiologic outcomes by noting the eradication of specific pathogens (e.g., reduction to below detection limits of *Blastocystis hominis*, *Dientamoeba fragilis*, or *Candida* spp.) at follow-up. No formal hypothesis testing was performed; results are reported as trends (e.g., number of patients improving vs. worsening) to inform future power calculations.

## 3. Results

### 3.1. Patient Clinical Characteristics

Feasibility outcomes showed that while 7 patients were enrolled, only 5 (71%) completed paired sampling; all 5 reported full adherence to 30-day supplementation; no serious adverse events were reported. Thus, five patients with confirmed CDI were finally included, ranging in age from 27 to 79 years (median 73 years; three males and two females). Four patients (80%) had at least one chronic comorbidity, reflected by a Charlson Comorbidity Index of 3 in three patients and 8 in one patient. The youngest patient (P5, 27 years) had no chronic illnesses or comorbid conditions (Charlson index 0), serving as a contrast case for purely iatrogenic dysbiosis, as opposed to the typical frailty of CDI-susceptible clinical populations (elderly, with significant chronic pathology). The most common underlying conditions were cardiovascular diseases (arterial hypertension in 3/5 cases, and one with ischemic heart disease and heart failure) and renal impairment (one with chronic kidney disease and one acute kidney injury), while one patient had type II diabetes mellitus and Parkinson’s disease as well. Despite the older cohort and its high comorbidity burden, all patients were functionally independent prior to CDI onset.

All cases were microbiologically confirmed CDI, with toxin-positive stool tests and acute diarrheal symptoms. Three patients (60%) presented with community-onset or healthcare-associated CDI, while two patients (40%) developed CDI during hospitalization. Disease severity was classified as mild in three cases and moderate in two cases (ATLAS score 1–3; a clinical severity scoring system); no patient developed fulminant disease. All patients received standard anti-CDI therapy with oral vancomycin for 10–14 days; the two moderate cases additionally received metronidazole. Hospital stays ranged from 0 to 10 days, according to disease severity. No systemic complications, surgical interventions, or CDI-related mortality occurred.

Following completion of antibiotic therapy, all patients underwent a 30-day course of a high-potency multi-strain probiotic as adjunct microbiome-directed therapy. By Day 30, all patients were clinically cured, with complete resolution of diarrhea. No CDI early recurrences were documented during the 30-day follow-up, and stool testing at follow-up confirmed the absence of *C. difficile* toxins in all patients. Broad stool analyses did not reveal acquisition of new enteric pathogens during the study period.

### 3.2. Global Microbiome Dynamics: Diversity and Dysbiosis

Despite inter-individual differences in age and comorbidity burden, baseline microbiome profiles shared a strikingly convergent pattern of severe dysbiosis. At baseline, all patients exhibited profound microbiome disruptions (collapsed diversity with *Proteobacteria* overgrowth and loss of obligate anaerobes), consistent with known patterns of post-antibiotic gut dysbiosis.

The Biodiversity Index (an ordinal 5-point diversity score) was markedly reduced in all cases (mean ± SD: 1.2 ± 0.45), with four patients scoring 1 (very low diversity) and only 2 in the remaining patient, indicating severely reduced species richness and evenness across the cohort. Consistently, the global Dysbiosis Index (a semi-quantitative, context-specific indicator of microbiota imbalance) was substantially elevated (45.2 ± 7.9), indicating pronounced deviation from a healthy reference microbiome. The longitudinal analysis of these indices revealed heterogeneous recovery trajectories, characterized by variable degrees of improvement or persistence of dysbiosis across patients.

**Figure 2:**
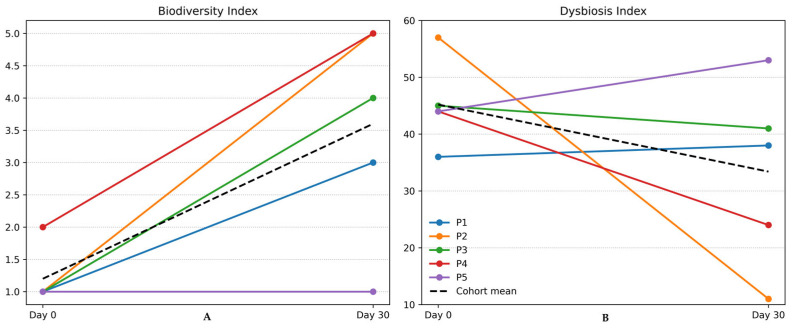
Longitudinal Changes in Biodiversity and Dysbiosis for Each Patient (P1–P5) from Baseline (Day 0) to Post-treatment (Day 30). Left (**A**): Biodiversity Index (ordinal scale 1–5; reflecting microbial richness and evenness) increased in 80% of patients by Day 30 (post-supplementation), indicating at least partial restoration of microbial diversity overall. Right (**B**): Dysbiosis Index (quantitative deviation score; lower values indicate a microbiota closer to a healthy reference—a downward slope indicates recovery) decreased in 60% of patients, reflecting individual variability in microbiota recovery. N.B.: Each colored line represents an individual patient, while the black dashed line denotes the cohort mean trajectory. P2 (orange) and P4 (red) demonstrated the most pronounced improvements, whereas P5 (purple) remained an outlier with persistently low diversity and high dysbiosis.

After the 30-day probiotic supplementation period, most patients showed a substantial increase in species richness. The mean Biodiversity Index rose from 1.2 to 3.6, indicating an overall trend toward a more eubiotic gut environment over the study period. As illustrated in Figure 2A (left panel), this aggregate improvement of alpha-diversity was driven by the complete restoration seen in P2 and P4 (maximal index of 5). Herein, despite advanced age and multimorbidity, P2 notably transitioned from a critically depleted state (index = 1) to a fully diverse ecosystem (index = 5), possibly suggesting a higher susceptibility to probiotic-aided restoration in this specific clinical context. Conversely, P5 (the youngest subject) showed a flat alpha-diversity trajectory, remaining stagnant at an index of 1. Overall, however, a substantial increase was observed during the supplementation period in alpha-diversity of the gut microbiome, namely an approximately threefold improvement.

Consistent with the gains in diversity, the overall Dysbiosis Index, a composite metric where scores >30 indicate a pathological state, provided a granular view of community health. Specifically, after 30 days, three out of five patients showed a decrease in dysbiosis score (P2, P3, P4; improvement), one remained roughly unchanged (P1; from 36 to 38), and one had an increased dysbiosis score (P5; worsened). Figure 2B (right panel) captures the particularly pronounced improvement trajectory of P2, whose score decreased from a critical 57 to a near-normal 11 (most substantial amelioration encountered). This contrasts sharply with P5, who exhibited a paradoxical deterioration (from 44 to 53), aligning with the absence of ecological recovery (lack of alpha-diversity improvement). Despite P1 showing no improvement and P5 slightly worsening, the mean Dysbiosis Index dropped from a severe 45.2 at baseline, to 33.4 at Day 30, amounting to a 26% improvement. This suggests an overall shift in the gut microenvironment towards a more eubiotic state over the 30-day period of probiotic supplementation, though the degree of improvement varied widely by individual.

However, given the semi-quantitative nature of the indices reported, these changes are interpreted directionally rather than as absolute effect sizes. In fact, the Dysbiosis Index, Biodiversity Index, F/B ratio, A/P ratio, and enterotype classification are all semi-quantitative measures. These parameters provide a general indication of microbiome imbalance but have limited reproducibility and cross-study comparability, so any categorical conclusions drawn from them should be made cautiously.

### 3.3. Integrated Taxonomic Changes at Phylum and Genus Levels

At baseline (D0), all five patients demonstrated pronounced intestinal dysbiosis following CDI and antibiotic exposure, characterized by convergent disturbances at both phylum and genus levels. Despite interindividual differences in age, comorbidity burden, and clinical context, baseline microbiome profiles converged at the level of higher-order ecological structure rather than identical taxonomic composition, reflecting a shared dysbiotic state characterized by collapse of obligate anaerobic communities (*Actinobacteria*- and butyrate-associated *Firmicutes*) and expansion of facultative and opportunistic *Proteobacteria*-dominated taxa.

#### 3.3.1. Phylum-Level Profiles

**Figure 3:**
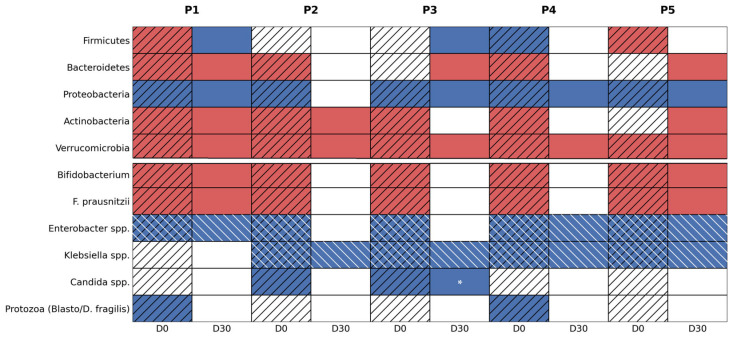
Integrated phylum- and genus-level taxonomic changes at baseline (Day 0) and after 30 days of probiotic therapy (Day 30). The figure presents a unified taxonomic matrix for all five patients (P1–P5), displaying major bacterial phyla (**upper** section), and selected commensal, opportunistic, and pathogen-related taxa at genus level (**lower** section), aligned to an identical patient–timepoint structure. Cell colors indicate deviation from laboratory reference ranges: red = decreased (↓), white = within reference range or absent (N), blue = increased or present (↑). Baseline (Day 0) samples are denoted by black diagonal hatching. For selected opportunistic and pathogenic taxa, white reverse cross-hatching applied to blue cells denotes a high pathogenic burden, defined qualitatively as either persistence of the same taxon across both timepoints or co-detection of multiple taxa within the same pathogenic group, rather than quantitative abundance. A white asterisk marks pseudo-persistent *Candida* spp., defined as continued fungal detection at Day 30 with a change in species composition between baseline and follow-up, rather than persistence of the identical species. Overall, we highlight patient-specific trajectories of structural dysbiosis, commensal recovery, pathogen persistence or clearance, and their relationship with microbiome reorganization.

As summarized in Figure 3, baseline phylum-level profiles were dominated by *Proteobacteria* expansion, accompanied by a reduction in *Actinobacteria* and frequent imbalance between *Firmicutes* and *Bacteroidetes*. From a ratio-based perspective, this constellation corresponds to a consistently decreased A/P ratio across all patients and, in a subset, an increased F/B ratio.

The A/P ratio is particularly informative in the post-antibiotic setting. *Actinobacteria*—driven largely by *Bifidobacterium* spp.—were consistently reported as decreased at baseline, while *Proteobacteria* were described as predominant or increased. The laboratory interpretations explicitly associate a decreased A/P ratio with antibiotic-induced dysbiosis and inflammatory mucosal states, reflecting both loss of colonization resistance and expansion of facultative pathogenic taxa capable of producing pro-inflammatory or mucosa-damaging metabolites—e.g., indoles, phenols, and hydrogen sulfide (H_2_S).

Alterations in the F/B ratio were more heterogeneous. Two patients (P2 and P4) exhibited a clearly increased F/B ratio at baseline, where *Firmicutes* clearly predominated over *Bacteroidetes*, a pattern the reports associate with altered energy extraction and metabolic orientation. In P1, *Bacteroidetes* were reduced without overt *Firmicutes* dominance, still contributing to functional imbalance and enterotype instability. Importantly, these ratios are interpreted qualitatively, in accordance with the diagnostic framework of the assay, rather than as reconstructed numerical values.

The mucin-associated phylum *Verrucomicrobia* (notably *Akkermansia muciniphila*) was absent or markedly reduced in all baseline samples, suggesting compromised mucosal barrier support across the cohort and foreshadowing incomplete recovery of mucin-associated functions at follow-up.

After 30 days of multi-strain probiotic therapy (D30), phylum-level remodeling remained heterogeneous and followed distinct, partially overlapping trajectories rather than a uniform recovery pattern (Figure 3). In light of the complexity of individual profiles and the small cohort assessed, we must note that each patient essentially displayed an individual trajectory, and any grouping into patterns is still preliminary. This being said, when changes were examined through the A/P ratio framework, two principal modes of partial A/P improvement emerged, alongside a pattern of persistent A/P dysbiosis.

A first improvement pattern was driven predominantly by a reduction in *Proteobacteria*. This was observed exclusively in P2, where *Proteobacteria* normalized from increased at baseline to within the reference range at D30 (↑→N), despite continued *Actinobacteria* depletion (↓→↓). In this patient, improvement of the A/P balance was therefore mediated by suppression of *Proteobacteria* rather than restoration of *Actinobacteria*.

A second improvement pattern was driven by recovery of *Actinobacteria*. This was observed in P3 and P4, where *Actinobacteria* increased from under to within the reference range at follow-up (↓→N), while *Proteobacteria* remained elevated (↑→↑). In these cases, partial A/P improvement was associated with restoration of *Actinobacteria*-associated taxa despite persistent *Proteobacteria* predominance.

In contrast, persistent A/P dysbiosis was observed in P1 and P5. P1 showed sustained *Proteobacteria* elevation and persistent *Actinobacteria* depletion (↑→↑ and ↓→↓), indicating no phylum-level improvement following intervention. P5 exhibited worsening of the A/P configuration, with *Proteobacteria* remaining elevated (↑→↑) and *Actinobacteria* declining from within reference range to decreased (N→↓), representing deterioration rather than recovery. Changes in the F/B ratio followed a similar heterogeneous pattern. Partial normalization or stabilization of the F/B relationship was observed in selected patients with partial A/P improvement, whereas persistence or redistribution of *Firmicutes*-dominant configurations was evident in patients with ongoing A/P dysbiosis, underscoring the absence of a uniform phylum-level recovery trajectory.

#### 3.3.2. Enterotype Behavior

**Figure 4:**
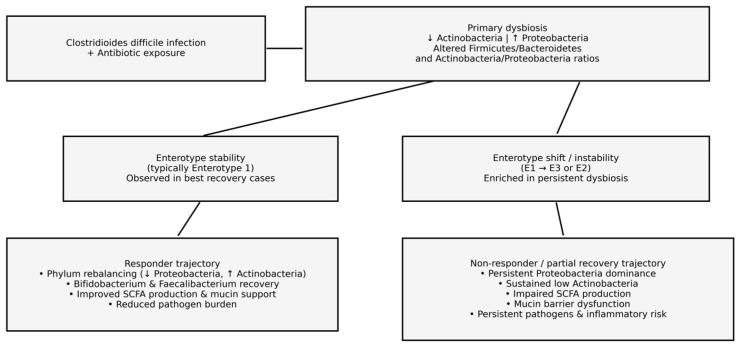
Conceptual model of gut microbiome recovery trajectories following *Clostridioides difficile* infection. The model summarizes the proposed sequence of microbiome alterations following CDI and antibiotic exposure. Initial dysbiosis is characterized by reduced *Actinobacteria*, increased *Proteobacteria*, and altered *Firmicutes/Bacteroidetes* (F/B) and *Actinobacteria/Proteobacteria* (A/P) ratios. From this dysbiotic state, remodeling may diverge into two broad trajectories. In a partial-recovery trajectory, improvements in higher-order structure (e.g., partial A/P improvement via *Proteobacteria* reduction and/or *Actinobacteria* recovery) may occur alongside variable restoration of beneficial taxa (e.g., *Bifidobacterium*) and incomplete recovery of key butyrate-associated genera (including *Faecalibacterium prausnitzii*), with selective reductions in pathogenic burden. In contrast, persistent dysbiosis is characterized by sustained *Proteobacteria* predominance with limited restoration of *Actinobacteria*-associated taxa and ongoing opportunistic pressure. Enterotype transitions (E1→E3 or E1→E2) may occur during follow-up and should be interpreted as an additional, non-redundant dimension of remodeling rather than a deterministic marker of recovery. N.B.: The figure represents a conceptual synthesis of observed taxonomic and functional patterns rather than a deterministic or patient-level classification model.

These structural changes were closely linked to enterotype behavior. As conceptualized in Figure 4, enterotype trajectories following probiotic therapy were heterogeneous and only partially aligned with phylum-level A/P remodeling. Although all patients exhibited Enterotype 1 (*Bacteroides*-dominated) profiles at baseline, consistent with Western dietary patterns and the post-antibiotic context, three patients demonstrated enterotype transitions by Day 30. Specifically, P1 and P3 shifted from Enterotype 1 to Enterotype 3 (*Ruminococcus*-dominated), while P5 transitioned from Enterotype 1 to Enterotype 2 (*Prevotella*-dominated). In contrast, enterotype stability was preserved in P2 and P4, both of whom remained within Enterotype 1 throughout follow-up.

Importantly, enterotype shifts were not restricted to patients with persistent A/P dysbiosis, as P3 exhibited *Actinobacteria*-driven partial A/P improvement despite transitioning to Enterotype 3. Conversely, enterotype stability was observed in both a *Proteobacteria*-driven A/P improver (P2) and an *Actinobacteria*-driven A/P improver (P4).

These shifts did not represent restoration of a healthy microbiome structure, but rather reorganization into alternative ecological equilibria, consistent with incomplete recovery of key commensal niches. In fact, enterotypes are increasingly being viewed as ecological attractors rather than fixed categories; accordingly, rapid shifts during convalescence should be interpreted as plasticity under perturbation (antibiotics, acute illness, and potentially dietary change) and thus may more likely reflect ecosystem instability, rather than uniform normalization. Therefore, collectively, our findings might actually indicate that enterotype instability reflects a distinct dimension of post-CDI microbiome remodeling that is related to, but not determined solely by, phylum-level A/P dynamics.

This divergence supports the interpretation that enterotype instability is a marker of impaired ecological recovery, rather than a universal feature of post-CDI microbiome remodeling. Enterotype transitions therefore emerged as a complementary, but non-redundant, descriptor of microbiome recovery trajectories, motivating subsequent functional and multivariate integration analyses. Even so, it is worth noting that all participants also received dietary advice that was not controlled, which serves as an important confounder. Thus, changes in dysbiosis indices or enterotypes during follow-up may partly reflect dietary influences rather than probiotic effects alone.

#### 3.3.3. Genus-Level Contextualization

The lower portion of Figure 3 contextualizes these phylum-level changes at the genus level. At baseline, all patients exhibited depletion of *Bifidobacterium* spp. and *Faecalibacterium prausnitzii*—two taxa repeatedly highlighted in the reports as central to SCFA production, mucosal integrity, and immune modulation.

Following probiotic therapy, partial re-emergence signals in one or both genera were observed in a subset of patients (P2–P4) and closely paralleled improvements in *Actinobacteria* representation and A/P ratio. In contrast, patients without phylum-level recovery remained depleted in these genera (P1 and P5), supporting the concept that genus-level restoration is constrained by higher-order taxonomic architecture.

Opportunistic and potentially pathogenic taxa, predominantly belonging to *Proteobacteria*, showed variable responses. While clearance of specific organisms was observed in some patients, others retained persistent or high pathogenic burden, particularly involving *Enterobacteriaceae*. Across the cohort, the reports explicitly flagged multiple facultative pathobionts, including *Enterobacter* spp., *Klebsiella* spp., *Acinetobacter* spp., *Serratia* spp., *Citrobacter* spp., *Proteus* spp., *Morganella* spp., *Haemophilus* spp., and *Pseudomonas* spp., whose trajectories were patient-specific and heterogeneous.

A clearance-dominant pattern was observed in P2, in whom a baseline multi-genera burden (*Acinetobacter*, *Enterobacter*, *Serratia*, and *Klebsiella*) contracted at Day 30 to a single residual genus (*Klebsiella*), consistent with partial restoration of colonization resistance. Similarly, P4 demonstrated selective clearance of *Pseudomonas* spp. and *Haemophilus* spp. between baseline and follow-up, while residual *Acinetobacter*, *Enterobacter*, and *Klebsiella* persisted, indicating incomplete but directional suppression of facultative *Proteobacteria*.

In contrast, a persistence pattern was evident in P1, in whom *Enterobacter* spp. were detected at both timepoints, and in P5, who exhibited a stable multi-genus *Enterobacteriaceae* profile (including *Citrobacter*, *Enterobacter*, *Klebsiella*, *Proteus*, and *Haemophilus*) across baseline and follow-up. Finally, P3 exhibited a mixed pattern by Day 30, characterized by normalization of *Enterobacter* spp. with persistence of *Klebsiella* spp., indicating selective rather than global suppression of facultative *Proteobacteria*; however, the raw reports additionally documented emergence of other opportunistic genera (including *Morganella* and *Proteus*, not visualized in Figure 3), consistent with increased genus-level pathogenic burden despite partial phylum-level remodeling. In Figure 3, high pathogenic burden is deliberately highlighted only when explicitly supported by the reports—either through multiplicity of taxa or persistence across both timepoints—thereby avoiding overinterpretation.

Collectively, these findings indicate that recovery after CDI is governed not merely by shifts in individual taxa but by restoration of higher-order microbial architecture, particularly (partial) normalization of the *Actinobacteria*–*Proteobacteria* balance and stabilization of enterotype structure. Given the well-established functional roles of these taxonomic groups in SCFA production, mucosal barrier maintenance, and suppression of opportunistic pathogens, the observed heterogeneity in taxonomic recovery is expected to translate into divergent functional outcomes, addressed in the following section.

#### 3.3.4. Pathogenic and Protozoal Clearance

Clearance and remodeling of pathogenic and protozoal taxa were observed during follow-up and are summarized in Figure 3. At baseline (D0), two patients (P1 and P4) demonstrated colonization with *Blastocystis hominis*, while P1 additionally showed borderline detection of *Dientamoeba fragilis*. At D30, none of these patients tested positive for *Blastocystis hominis* or *Dientamoeba fragilis*, indicating complete clearance of these protozoa across the cohort. In the remaining patients (P2, P3, and P5), the multiplex protozoal panel was negative at baseline and remained negative at follow-up. No other intestinal protozoal pathogens (including *Giardia lamblia*, *Cryptosporidium* spp., *Entamoeba histolytica*, or *Cyclospora cayetanensis*) were detected at either timepoint.

*Candida* spp. dynamics further illustrated the complexity of genus-level remodeling, being patient-specific and species-dependent. At baseline, *Candida albicans* was detected in P2 and P3, with concurrent *Candida glabrata* detected in P2. By D30, P2 demonstrated complete fungal clearance, with no yeasts detected. In contrast, P3 exhibited a species transition rather than eradication: *Candida albicans* was no longer detected, while alternative species (*Candida krusei* and *Candida lusitaniae*) emerged at low abundance. This pattern represents pseudo-persistence via species replacement rather than persistence of the original fungal species and is explicitly denoted in Figure 3 (white asterisk). No yeasts were detected in the remaining patients at either timepoint.

All patients were negative for *Clostridioides difficile* toxin genes at D30, and no early recurrence of CDI occurred during the observation period. Importantly, no patient acquired new intestinal protozoal pathogens during follow-up. However, in contrast to the uniform clearance observed for protozoa, opportunistic bacterial pathogens exhibited heterogeneous trajectories: several *Proteobacteria* genera cleared between baseline and follow-up (e.g., *Pseudomonas* spp. and *Haemophilus* spp. in P4; *Acinetobacter* spp. and *Serratia* spp. in P2), whereas other patients demonstrated persistence (P1, P5) or expansion (P3) of facultative pathobiont profiles.

Collectively, these findings indicate that, alongside partial bacterial community remodeling, probiotic therapy coincided with clearance of protozoal colonization and resolution or restructuring of opportunistic fungal carriage. In contrast, clearance of opportunistic bacterial taxa was variable and strongly patient-dependent, contributing to residual pathogenic burden in selected individuals despite clinical recovery.

### 3.4. Functional Implications of Incomplete Taxonomic Recovery

**Figure 5:**
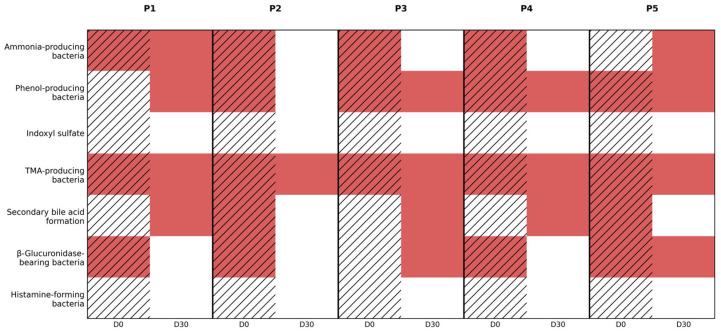
Functional and metabolite-related microbiome markers at baseline (Day 0) and after 30 days (Day 30). Matrix summarizing non-taxonomic functional markers reported by the microbiome interpretation (ammonia-producing bacteria, phenol-producing bacteria, indoxyl sulfate, TMA-producing bacteria, secondary bile acid formation, β-glucuronidase-bearing bacteria, and histamine-forming bacteria) across patients P1–P5. Red indicates increased/unfavorable status, while white indicates normal/unremarkable status. D0 samples are denoted by black hatching. The figure visualizes persistent versus improving metabolic signatures of dysbiosis after clinical recovery.

Beyond taxonomic restructuring, longitudinal microbiome profiling revealed persistent and patient-specific alterations in inferred metabolic and functional capacities following CDI and antibiotic exposure. These functional signatures, summarized in Figure 5, are derived from the abundance of bacterial taxa known to harbor specific metabolic pathways and explicitly represent inferred (predicted) functions rather than direct fecal metabolite measurements. In other words, all metabolic and functional results (e.g., SCFA production or TMA reduction) are inferred from the 16S taxonomic data rather than measured directly via metabolite assays. Accordingly, functional categories reported by the assay reflect inferred metabolic potential, based on the presence of recognized producer taxa, rather than confirmed metabolite concentrations.

At D0, all patients exhibited an unfavorable functional profile characterized by increased abundance of ammonia-producing and phenol-producing bacteria, consistent with a dysbiotic state dominated by facultative anaerobes and proteolytic taxa. Specifically, ammonia-producing capacity is primarily associated with proteolytic members of *Proteobacteria* and certain *Firmicutes*, including *Enterobacteriaceae* (e.g., *Enterobacter*, *Klebsiella*, *Proteus*) and selected *Clostridium* species. Phenol-producing potential similarly reflects enrichment of aromatic amino acid-fermenting taxa, again dominated by *Enterobacter*, *Klebsiella*, *Proteus*, and related opportunistic genera. The uniform elevation of these categories at baseline across all patients indicates a shared shift toward protein fermentation and nitrogenous waste generation following antibiotic exposure.

After 30 days of probiotic therapy (D30), partial attenuation of these proteolytic functional signatures was observed in a subset of patients, while others retained an unfavorable profile. Specifically, inferred ammonia-producing capacity showed partial normalization or attenuation in P2, P3, and P4, coinciding with partial suppression of *Proteobacteria* at the phylum/genus level. In contrast, P1 and P5 demonstrated persistent elevation of ammonia-associated taxa, paralleling sustained *Proteobacteria* predominance. Phenol-producing signatures followed a similar heterogeneous pattern, indicating that proteolytic metabolic pressure was not uniformly resolved despite clinical recovery.

Markers related to indole and uremic toxin metabolism, represented herein as indoxyl sulfate-associated signatures, were largely unremarkable or normalized at both timepoints in most patients. Indoxyl sulfate production is linked to tryptophan-metabolizing taxa, including *Escherichia coli* and selected *Clostridium* species. The absence of prominent indoxyl sulfate-associated signals suggests that, despite marked dysbiosis, tryptophan-derived uremic toxin pathways were not a dominant functional abnormality in this cohort. This finding should be interpreted cautiously, as inferred pathway absence does not exclude transient/low-level metabolite production below the assay’s resolution.

In contrast, TMA-producing bacterial signatures were increased at baseline in most patients, reflecting expansion of taxa capable of choline and carnitine metabolism. This functional category is primarily associated with *Proteobacteria* and select *Firmicutes*, including *Enterobacteriaceae*, *Desulfovibrio*, and certain *Clostridia*. At D30, TMA-associated functional signals improved in P2 and P4, consistent with partial reduction in *Proteobacteria* burden, but persisted in P1, P3, and P5, indicating resistance of cardiometabolic risk–associated microbial pathways to short-term intervention in these individuals, particularly in the context of incomplete structural recovery.

Baseline profiles also frequently indicated increased representation of bacteria associated with secondary bile acid formation, a functional capacity largely attributed to *Firmicutes* within *Clostridium* clusters involved in bile acid dehydroxylation. Following probiotic therapy, secondary bile acid-associated signatures demonstrated limited improvement, remaining elevated in most patients despite partial phylum-level remodeling. This persistence suggests incomplete reconstitution of bile acid homeostasis and continued exposure to potentially cytotoxic bile acid derivatives.

Similarly, β-glucuronidase-bearing bacteria were increased or borderline at baseline and showed only modest normalization at D30. β-glucuronidase activity is commonly associated with *Enterobacteriaceae* (e.g., *Escherichia*, *Klebsiella*) and selected *Firmicutes*. Given the role of microbial β-glucuronidase in enterohepatic recirculation of hormones, xenobiotics, and toxins, these findings suggest persistent functional vulnerability to reactivation of conjugated compounds despite clinical recovery.

In contrast to other functional domains, histamine-forming bacteria were largely within the reference range at both timepoints across the cohort. Histamine production is associated with taxa such as *Morganella*, *Enterobacter*, and selected *Lactobacillus* species. The absence of consistent elevation suggests that histamine-mediated dysregulation did not represent a major component of post-CDI dysbiosis in these patients.

Collectively, these findings demonstrate that functional recovery lagged behind partial taxonomic remodeling, with persistent inferred metabolic signatures of dysbiosis evident in several patients at D30. Improvements were selective rather than global, and closely tracked higher-order taxonomic features, particularly persistence of *Proteobacteria* and incomplete restoration of *Actinobacteria*-associated taxa. Importantly, clinical symptom resolution occurred despite ongoing unfavorable functional signatures in some patients, underscoring the dissociation between short-term clinical recovery and full ecological or metabolic normalization of the gut microbiome. Taken together, these preliminary functional patterns suggest that higher-order structural improvement does not necessarily translate into rapid normalization of metabolic potential, motivating the use of multivariate integration to capture global recovery trajectories in the subsequent analysis.

### 3.5. Multivariate Integration and Global Microbiome Recovery Trajectories

To explore overall microbiome restructuring at the individual level, principal component analysis (PCA) was applied to four selected variables: Biodiversity Index, Dysbiosis Index, *Firmicutes* relative abundance, and *Proteobacteria* relative abundance (10 samples: 5 patients × 2 time points). All variables were z-score standardized prior to PCA to avoid scale-driven bias. These variables were derived from laboratory-reported categorical or semi-quantitative indices rather than absolute abundance values, and PCA therefore reflects relative ecological positioning rather than precise quantitative distances.

To ensure robustness given the limited sample size, PCA was restricted to these four orthogonal variables capturing ecological (biodiversity), clinical (dysbiosis index), and structural (*Firmicutes* and *Proteobacteria* relative abundances) dimensions of the microbiome. Additional taxonomic or functional variables were deliberately excluded to avoid redundancy and overfitting.

**Figure 6:**
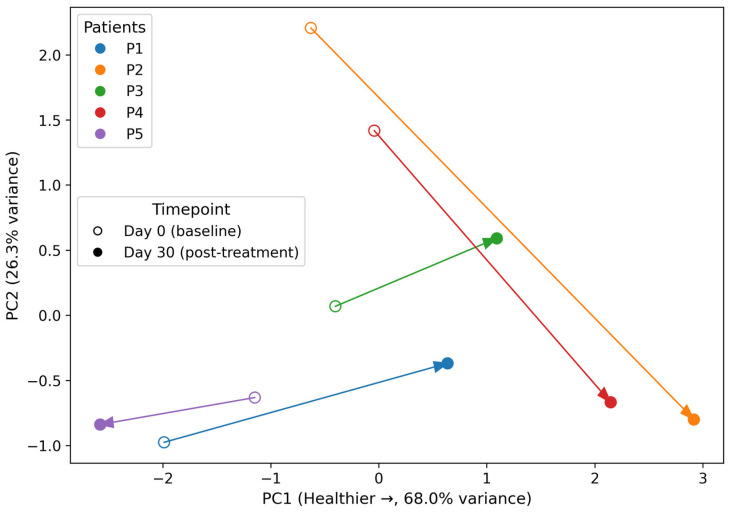
Principal Component Analysis (PCA) Ordination of Overall Microbiome Profiles Before vs. After Treatment. Each arrow represents one patient’s trajectory in principal component space from Day 0 (arrow tail) to Day 30 (arrow head). PC1 explained 67.96% of total variance, while PC2 explained 26.26%, for a cumulative variance of 94.22%. PC1 separates dysbiotic vs. recovered microbiomes: Day 0 samples (hollow circles at arrow tails) all cluster on the left (negative PC1), whereas most Day 30 samples (solid circles at arrow heads) shifted rightward into positive PC1, indicating more normalized profiles. Patients 2 (orange) and 4 (red) show the largest shifts, moving into a region associated with higher diversity, lower dysbiosis, and greater abundances of beneficial taxa. Patients 1 (blue) and 3 (green) showed more modest shifts—their Day 30 positions moved somewhat toward normal, but still remained intermediate (retaining some residual dysbiosis). Patient 5 (purple) notably moves left/down—reflecting the single outlier who did not improve. Overall, most patients demonstrated displacement toward a region of ordination space associated with healthier microbiome profiles after probiotic therapy. N.B.: PCA is shown as a descriptive visualization only, given the limited sample size, and does not represent a robust statistical model of microbiome recovery.

PCA loadings derived from the standardized data showed positive contributions of Biodiversity Index and *Firmicutes* and negative contributions of Dysbiosis Index and *Proteobacteria* to PC1, confirming that PC1 represents a global microbiome health axis. The variance explained by PC1 (67.96%) and PC2 (26.26%) corresponds to the direct PCA output of the standardized dataset and was not post hoc adjusted, yielding a cumulative variance of 94.22%. These trajectories are visualized in Figure 6, which demonstrates marked rightward shifts (toward healthier microbiome states) in four patients, with minimal or adverse displacement in patient P5.

Importantly, given the very small number of observations, PCA is presented solely as a descriptive visualization of within-subject displacement over time and should not be interpreted as a stable inferential model or as evidence of clustering or separable recovery states. Therefore, these trajectories (Figure 6) serve to contextualize individual longitudinal changes rather than to define generalizable patterns.

### 3.6. Composite Abnormality Burden

**Figure 7:**
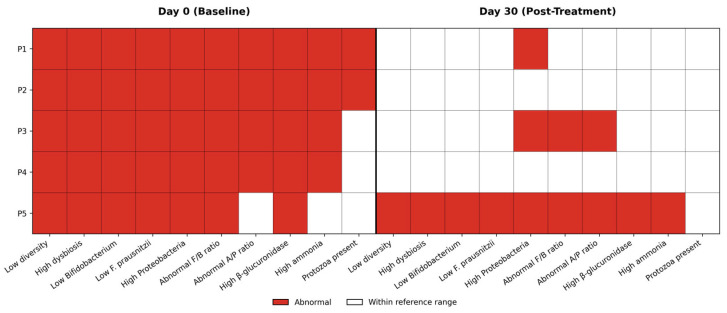
Binary Abnormality Heatmap of Key Microbiome Parameters Per Patient. Each cell indicates the presence (red) or absence (white) of an abnormal finding for a given parameter in a given patient. Baseline (Day 0) results are shown on the left and follow-up (Day 30) on the right for each patient (P1–P5), with a vertical divider between them. The heatmap encompasses multiple domains—diversity, specific genera, and metabolic markers—and highlights that most abnormalities present at Day 0 (red squares) shifted to normal by Day 30 (white squares) in the majority of patients, underscoring the broad reduction in dysbiosis-associated findings after probiotic therapy, while also indicating which issues (e.g., persistent *Proteobacteria* overgrowth in P5) did not fully resolve.

A binary abnormality heatmap (Figure 7) integrates diversity, taxonomic, and metabolic parameters at the patient level. At baseline, all patients exhibited multiple concurrent abnormalities across domains. By Day 30, the abnormality burden decreased substantially, though residual abnormalities persisted in selected domains and patients.

The composite abnormality burden represents the cumulative count of parameters classified as abnormal (outside laboratory reference ranges or qualitatively flagged as unfavorable), summarized at the individual patient level for each timepoint, across three domains: (1) global diversity and dysbiosis indices; (2) selected taxonomic features (including key commensal deficits and opportunistic taxa); and (3) inferred functional or metabolic risk markers. Each parameter is treated as a binary state (abnormal vs. non-abnormal), without weighting, yielding an integrated snapshot of dysbiosis severity rather than a quantitative severity score. This approach intentionally prioritizes breadth of dysregulation over magnitude, capturing the coexistence of multiple ecological and functional perturbations within the same patient. As such, a reduction in abnormality burden reflects contraction of dysbiosis across domains, whereas persistence indicates multi-dimensional ecological instability.

At baseline, all patients exhibited high composite abnormality burdens, consistent with severe post-antibiotic dysbiosis. By Day 30, most patients showed a reduction in the number of abnormal parameters, reflecting partial ecological recovery. However, P5 retained a high abnormality burden driven by persistent *Proteobacteria* overgrowth and unresolved functional abnormalities, while P1 demonstrated limited reduction, underscoring heterogeneity in recovery trajectories.

Collectively, the composite abnormality burden provides an integrative framework linking taxonomic disruption, functional risk signatures, and clinical recovery, highlighting that symptom resolution may occur despite persistence of multi-domain microbiome abnormalities.

## 4. Discussion

In this pilot study, a 30-day course of a 10-strain probiotic (OB10) was associated with notable, yet highly variable, gut microbiota changes across five patients recovering from CDI. We observed a clear increase in microbial diversity and partial shifts in taxonomic composition during the supplementation period. Overall bacterial alpha-diversity increased in four of five subjects (reflected by higher biodiversity indices at 30 days), indicating partial microbiome recovery rather than complete normalization. These observations are consistent with prior reports describing the capacity of multi-strain probiotics to modulate community richness and compositional balance following antibiotic-induced dysbiosis [63,64]. However, in the absence of a control group, it is not possible to determine to what extent the observed microbiome changes exceed the expected trajectory of spontaneous post-antibiotic recovery. Furthermore, the concurrent dietary and lifestyle recommendations provided to participants (for e.g., a high-fiber diet to promote commensal regrowth) during the study represent an additional, uncontrolled/untracked, potentially important confounding factor, that may have significantly influenced microbiome remodeling independently.

Notably, most participants (four out of five) were older adults with substantial comorbidity burdens, a context in which age-associated microbiome features (“inflammaging”) may accentuate post-antibiotic dysbiosis. Aging is commonly associated with lower alpha-diversity, depletion of key obligate anaerobes (including *Bifidobacterium* spp. and butyrate-associated taxa such as *Faecalibacterium prausnitzii*), and relative expansion of facultative anaerobes (particularly members of the *Enterobacteriaceae* family) [65,66]. This background state may also influence both baseline profiles and the pace and completeness of recovery. Within this context, the observed expansion of beneficial taxa (including *Bifidobacterium* and *Faecalibacterium*) alongside reductions in selected opportunistic organisms suggests partial reconstitution of colonization resistance. Such patterns are clinically relevant, as increased microbial diversity and enrichment of commensal anaerobes are associated with enhanced resistance to pathogen overgrowth and lower inflammatory tone [65].

The increase in the Biodiversity Index from very low (scores 1–2) to moderate/high (3–5) in 80% of patients is notable. Loss of diversity is a hallmark of CDI-related dysbiosis [66], and low diversity correlates with greater risk of recurrent infection and poorer gut health outcomes. The observed rebound in diversity suggests a re-assembly of the complex microbial network needed for a resilient ecosystem. This finding is consistent with prior reports that OB10 can restore and maintain bacterial diversity in the gut [67]. It is also reminiscent of the effects reported for FMT, which is known to rapidly increase microbiome diversity in CDI patients [68]. While it was beyond the scope of our study to directly compare probiotics to FMT, the alpha-diversity gains achieved longitudinally suggest that, at least in principle, a high-potency multi-strain probiotic intervention might be capable of achieving some of the same microbiome restoration benefits as seen after FMT, though likely to a lesser degree and at a slower pace, yet in a safer and more controlled manner. However, given the current uncontrolled pilot design and short follow-up, no conclusions regarding comparative effectiveness, durability of recovery, or clinical equivalence between probiotics and FMT can be inferred. Consequently, probiotics should not be viewed as replacements for FMT, but rather as potential adjuncts in early recovery.

In terms of community composition, our patients’ baseline profiles were characterized by the classic post-antibiotic pattern: depletion of keystone commensals (e.g., *Faecalibacterium* and *Bifidobacterium*) and proliferation of *Proteobacteria* (e.g., *Enterobacteriaceae*) [69]. This corresponds to a consistently decreased A/P ratio and a frequently altered F/B relationship. After probiotic treatment, we documented heterogeneous but directional remodeling of these imbalances. Three out of five patients (P2–P4) exhibited partial A/P improvement, including one case driven predominantly by *Proteobacteria* reduction and two cases driven by *Actinobacteria* recovery. F/B partial normalization or stabilization was also observed in a subset of patients, whereas persistence of dysbiosis-associated configurations remained evident in others. Although not all patients fully normalized at the phylum level, the direction of change was toward eubiosis in most cases. These results support the idea that multi-strain probiotics may help shift the gut ecosystem towards a healthier state, even if complete normalization may require a longer time or additional measures.

One plausible ecological explanation for *Proteobacteria* predominance after antibiotic exposure is the so-called “oxygen hypothesis”: mucosal inflammation and reduced butyrate availability may increase epithelial oxygen and nitrate flux into the lumen, favoring facultative anaerobes (including *Proteobacteria*) over strict anaerobes [70]. Although this study did not measure host permeability or inflammatory biomarkers directly, this framework is consistent with the observed co-occurrence of *Proteobacteria* expansion and depletion of butyrate-associated taxa.

One interesting observation was the heterogeneity of response. While some patients exhibited exceptional improvements (e.g., a >4-fold diversity increase and dysbiosis index drop from 57→11 in one case), one patient showed minimal changes and another even slight worsening in certain parameters. This variability could stem from differences in baseline dysbiosis severity or other host factors. For example, the single instance of worsening Dysbiosis Index was seen in P5, which had the mildest microbiome disruption at baseline (low diversity but nearly normal phylum proportions), hypothetically leaving fewer “open” niches for the probiotic to occupy. Conversely, patients with the most severe initial dysbiosis (e.g., P2) experienced the largest gains, perhaps because the probiotic could more readily colonize an ecosystem with many additional vacant niches. Differences in antibiotic pretreatment (e.g., vancomycin alone versus metronidazole + vancomycin) might have also, theoretically, influenced these outcomes, although our sample size is too limited to draw conclusions.

Moreover, some patients experienced shifts in overall community type (*enterotype*) during recovery. Three subjects transitioned from a baseline *Bacteroides*-dominated enterotype (Enterotype 1) to a different enterotype by Day 30 (two to *Ruminococcus*-dominated Enterotype 3, and one to *Prevotella*-dominated Enterotype 2). To a point, the shift to Enterotype 3 (*Ruminococcus*-dominant) observed in two patients may partially reflect the high-fiber dietary advice provided, as *Ruminococcaceae* are known primary degraders of resistant starch. Unfortunately, dietary advice was not strictly monitored (e.g., food diaries were not collected), preventing precise correlation between fiber intake and Enterotype shifts. Even so, such enterotype instability over a short interval is unusual and indicates that the gut ecosystem was reorganizing into an alternative equilibrium rather than simply reverting to its pre-infection state. Notably, these shifts were not restricted to patients with incomplete phylum-level recovery; for instance, one patient (P3) showed an improved A/P ratio yet still switched enterotype. Enterotype changes may thus represent an additional, independent dimension of post-CDI microbiome remodeling, highlighting that community structure can remain in flux even as certain compositional parameters improve.

Importantly, no CDI early recurrences occurred in our cohort during the 30-day follow-up period, and all patients remained toxin-negative at Day 30, but given the lack of a control group, we cannot attribute this outcome to the probiotic. In parallel, protozoal colonization detected at baseline (including *Blastocystis hominis* and borderline *Dientamoeba fragilis* in one patient) was uniformly cleared, whereas trajectories of opportunistic bacterial taxa were heterogeneous, with clearance, persistence, or expansion depending on the individual patient. These findings indicate that, while early post-CDI convalescence in this cohort was clinically uncomplicated, microbiological recovery was incomplete and domain-specific, rather than uniform.

The absence of early recurrence should therefore be interpreted in the context of partial microbiome remodeling rather than definitive recurrence prevention. Persistent dysbiosis—particularly ongoing *Proteobacteria* predominance and residual inferred functional abnormalities—remained evident in several patients despite symptom resolution, underscoring the known dissociation between short-term clinical recovery and ecological normalization of the gut microbiome. Given that CDI recurrence is strongly associated with persistent dysbiosis and impaired colonization resistance [71], the observed early stability may reflect a transitional state of recovery, rather than durable protection.

Within these limitations, our findings support the concept that targeted, multi-strain probiotic supplementation may contribute beneficially to microbiome remodeling during early post-CDI recovery, particularly by promoting diversity gains and partial restoration of beneficial commensal niches. Such formulations combine strains with complementary ecological functions—including SCFA production, competitive exclusion of opportunistic taxa, and immune modulation—mechanisms that are thought to be more effective for complex antibiotic-induced dysbiosis than using a single-strain approach [72]. However, the persistence of opportunistic bacteria in several patients highlights that probiotic therapy alone may be insufficient to fully normalize the microbiome within 30 days, and that sustained recovery may require longer duration, adjunct dietary strategies, or alternative microbiota-directed interventions.

Comparison with existing literature demonstrates partial alignment with prior observations regarding multi-strain probiotic use in CDI. Several studies have reported that probiotic formulations containing *Bifidobacterium* and *Lactobacillus* species may reduce ADD and, in selected contexts, CDI recurrence, when used adjunctively to standard therapy [11,27]. In a small case series, results described the absence of expected recurrences following administration of a multi-strain probiotic formulation closely related to OB10 [37]. While the present study was not designed to assess recurrence prevention and follow-up was limited to 30 days, the absence of early CDI relapse in a cohort with marked baseline dysbiosis is consistent with reports describing early clinical stability during probiotic-supported post-CDI recovery.

At the microbiological level, however, our findings extend prior literature by highlighting the heterogeneous and domain-specific nature of recovery. Although reductions in selected opportunistic Gram-negative genera (e.g., *Enterobacter*) were observed in some patients, persistence of other taxa (notably *Klebsiella*) and emergence of additional opportunistic genera in selected cases underscore that probiotic-associated remodeling does not equate to uniform pathogen eradication within a short timeframe. These observations emphasize that probiotics may facilitate partial ecological rebalancing rather than comprehensive normalization, and that persistent dysbiosis may require longer intervention duration, dietary modulation (e.g., prebiotic fiber supplementation to support butyrate-producing commensals), or alternative microbiota-directed strategies in refractory cases.

Recent advances in microbiome-based therapeutics further contextualize these findings. Defined microbial consortia and fecal microbiome-derived products—such as enema-administered microbiome suspensions and oral spore-based formulations (e.g., SER-109)—have demonstrated efficacy in preventing recurrent CDI in randomized trials [73], validating the principle that restoration of commensal diversity can disrupt the recurrence cycle. Specifically, in a Phase III trial (ECOSPOR III), SER-109 (comprising purified *Firmicutes* spores) was superior to placebo in preventing CDI relapse (12% vs. 40% recurrence at 8 weeks) [73], leading to its FDA approval in 2023. Compared with these approaches, multi-strain probiotics represent a more accessible and lower-risk strategy for promoting early microbiome recovery, although their effects appear more gradual and less comprehensive than FMT or defined live biotherapeutics. This positions probiotics not as replacements for established microbiome-based therapies, but as adjunctive modulators of recovery, particularly in the early post-infection phase.

Although no serious adverse events were observed in our cohort, probiotic administration should be approached cautiously in vulnerable populations, particularly in severely immunocompromised, critically ill, or central line-dependent patients. Rare cases of probiotic-associated bacteremia or fungemia have been reported in such settings. Therefore, while multi-strain probiotics appear well tolerated in clinically stable individuals, careful patient selection remains essential.

Taken together, these observations support a key interpretative model in which early post-CDI microbiome recovery proceeds through partial, non-linear, and patient-specific remodeling rather than uniform restoration. Probiotic-supported recovery appears to act primarily at the level of higher-order ecological structure—manifested as improvements in diversity, partial rebalancing of the A/P axis, and selective re-emergence of beneficial taxa—while genus-level and inferred functional normalization often lags behind. Enterotype transitions observed during follow-up further indicate that recovery frequently involves reorganization into alternative microbial equilibria rather than reversion to a pre-infection state. Within this framework, early clinical stability and absence of short-term recurrence may coexist with residual dysbiosis, reflecting a transitional phase in which colonization resistance is improving but not yet fully re-established. This model reconciles the observed dissociation between symptom resolution, taxonomic recovery, and inferred metabolic normalization, and underscores the importance of evaluating post-CDI recovery using integrative, multivariate approaches that capture structural, functional, and ecological dimensions of the microbiome, rather than relying on single taxa or short-term clinical endpoints alone.

## 5. Limitations and Future Directions

Despite these descriptive findings, several important limitations necessarily temper interpretation and generalizability. First, the very small sample size (five patients with paired data) and the absence of a placebo or untreated control group preclude definitive causal inference regarding the specific effects of probiotic supplementation. Although the direction and magnitude of microbiome changes—particularly in patients with marked baseline dysbiosis— may appear inconsistent with complete reliance on spontaneous recovery alone, the present design precludes differentiation between the degree of supplementation-associated changes versus the natural resilience and rebound of the post-CDI microbiome. Moreover, all participants received dietary and lifestyle recommendations; adherence was not formally monitored, and thus diet-related changes may constitute a significant uncontrolled confounder that may have also contributed to microbiome remodeling, further limiting attribution to probiotic supplementation specifically. Future initiatives should incorporate structured dietary recording (e.g., food diaries or standardized intake assessments) to better differentiate diet-associated microbiome shifts from supplementation-related changes. Indeed, larger, controlled, longitudinal studies will be required to distinguish intervention-associated effects from time-dependent recovery and to quantify clinically meaningful effect sizes. Likewise, our PCA results are shown only for exploratory, descriptive insight given the extremely small sample size. These trajectories should not be viewed as inferential or generalizable findings.

Second, microbiome characterization relied on a targeted 16S rRNA gene sequencing-based diagnostic panel with semi-quantitative reporting (normal/low/high) rather than deep shotgun metagenomic sequencing. While this approach robustly captures higher-order taxonomic shifts and clinically relevant microbial groups, it constrains functional interpretation to inferred metabolic potential derived from 16S-based taxonomic profiling, which has limited species- and strain-level resolution and does not permit direct metabolomic quantification. As a result, strain-level engraftment of probiotic organisms, shifts in unmeasured taxa, and precise quantitative reconstruction of F/B and A/P ratios could not be assessed. Furthermore, the use of semi-quantitative commercial diagnostic reporting limits comparison with larger preexisting studies using raw metagenomic data, as these semi-quantitative indices are context-specific and not universally generalizable. In addition, this study did not include direct host inflammatory or permeability readouts (e.g., fecal calprotectin, zonulin) or untargeted metabolomic profiling of SCFAs and/or bile acids. Incorporating these biomarkers in future controlled longitudinal studies would allow closer linkage between taxonomic restructuring, inferred functional potential, and clinically relevant mucosal and metabolic endpoints. Future initiatives integrating shotgun metagenomics, metabolomics, and host inflammatory biomarkers are therefore strongly warranted to enable a more comprehensive evaluation of microbial and host-level recovery.

Third, the 30-day follow-up period captures only the early phase of post-CDI convalescence. As suggested by our results, microbiome recovery appears to be non-linear and patient-specific, with some individuals showing partial improvement and others exhibiting persistent or newly emerging dysbiosis features. Extended longitudinal monitoring is therefore essential to determine whether early improvements consolidate into durable ecological stability, whether residual dysbiosis resolves spontaneously, or whether additional or prolonged interventions are required. Longer follow-up would also clarify the durability of protozoal clearance and the long-term behavior of opportunistic bacterial taxa.

Fourth, while all patients achieved clinical resolution and no CDI early recurrences occurred during the short follow-up window, this study was not designed or powered to establish links between microbiome changes and clinical outcomes such as recurrence prevention. The dissociation observed between symptom resolution and incomplete microbiome normalization underscores the need for future studies that explicitly integrate microbiome metrics with longer-term clinical endpoints.

Finally, the pronounced heterogeneity in recovery trajectories highlights the importance of personalized microbiome-based therapeutic strategies. Baseline microbiota configuration—including diversity, A/P balance, and enterotype—likely influences responsiveness to a given probiotic formulation. Future work should therefore explore stratification approaches based on initial dysbiosis patterns to guide tailored probiotic or synbiotic regimens. For example, individuals with marked depletion of butyrate-producing taxa may benefit from concurrent prebiotic supplementation to support functional restoration, whereas patients with *Firmicutes*-dominant configurations may require alternative strategies or longer intervention durations. Developing such precision approaches, guided by microbiome profiling and integrative biomarkers, represents a key direction for translating microbiome research into effective post-CDI rehabilitation protocols.

## 6. Conclusions

This pilot study provides a detailed, integrative characterization of early gut microbiome recovery following CDI in patients receiving adjunctive multi-strain probiotic supplementation (OB10). Using longitudinal taxonomic, functional, and multivariate analyses, we observed that post-CDI recovery is marked by partial, heterogeneous, and non-linear microbiome remodeling, rather than uniform restoration of a pre-infection state. Improvements in microbial diversity and higher-order structural features—such as partial rebalancing of the A/P axis—were observed in most patients, while genus-level composition, inferred metabolic functions, and opportunistic taxa exhibited variable and patient-specific trajectories.

Importantly, clinical symptom resolution and absence of short-term recurrence occurred despite persistent ecological and inferred functional abnormalities in several individuals, underscoring the dissociation between early clinical stability and incomplete microbiome normalization. Enterotype transitions and residual dysbiosis further suggest that post-CDI convalescence often involves reorganization into alternative microbial equilibria, rather than simple reversal of antibiotic-induced damage.

Within these constraints, our preliminary findings support the concept that multi-strain probiotics may contribute beneficially to early-stage microbiome remodeling after CDI, particularly by facilitating diversity gains and partial restoration of beneficial commensal niches—though causation cannot be confirmed in our design. However, the persistence of dysbiosis in some patients highlights that probiotic supplementation alone is unlikely to fully normalize the gut ecosystem within a short timeframe. Furthermore, more rigorous analyses must validate the extent of probiotic supplementation benefits, as opposed to natural rebound and/or adjunct dietary measures.

Taken together, our findings should be interpreted as preliminary and hypothesis-generating, requiring validation in larger, controlled cohorts with formal statistical testing. This study emphasizes the need to conceptualize post-CDI recovery as a dynamic, multicomponent ecological process, best captured through integrative taxonomic and functional frameworks rather than isolated metrics or short-term clinical endpoints. These data provide a descriptive foundation for future controlled studies aimed at defining how microbiome-directed interventions can be optimized, personalized, and combined with complementary strategies to support durable ecological recovery following CDI.

## Figures and Tables

**Table 1:** Key Gut Microbiome Parameters—Definitions and Clinical/Functional Significance.

	Parameter Definition and Significance
Biodiversity Index	Richness and evenness of the gut microbiome was quantified by the Shannon diversity index and converted into a semi-quantitative score ranging from 1 (very low diversity) to 5 (high diversity). ↑ diversity indicates a more resilient ecosystem with protection against opportunistic overgrowth; ↓ diversity reflects an imbalanced microbiota, often seen after antibiotics or in disease states [50].
Dysbiosis Index	A calculated numerical score reflecting the overall degree of deviation from a healthy reference microbiota profile. The scale ranges from >10 (normal) to higher values (>40) in severe dysbiosis. ↑ scores indicate greater microbiome disruption and instability; ↓ scores indicate recovery and a shift toward a eubiotic state. Note: The Dysbiosis Index is a proprietary algorithm of the analyzing laboratory (Biovis Diagnostik), derived from the relative abundance of key indicator taxa.
Enterotype	Baseline community type classified by dominant genera for each sample. Enterotype 1: *Bacteroides*-dominant (associated with protein and fat metabolism); Enterotype 2: *Prevotella*-dominant (excels in carbohydrate fermentation); Enterotype 3: *Ruminococcus*-dominant (adept at fiber degradation) [51]. Enterotypes are often stable in healthy adults, but increasing evidence suggests they behave as ecological attractors along a compositional gradient and may exhibit short-term plasticity after perturbations (e.g., antibiotics, acute illness, or major dietary change); therefore, rapid enterotype shifts in this study were interpreted as markers of ecosystem instability/reorganization rather than definitive restoration [51].
F/B Ratio	Indicator of global microbiota balance in our analysis. ↑ F/B ratio (*Firmicutes* ≫ *Bacteroidetes*) has been linked to obesity and IBS, possibly due to *Firmicutes*’ enhanced capacity to ferment indigestible carbohydrates into absorbable SCFAs, i.e., with greater caloric harvest and gas production, potentially exacerbating symptoms like bloating. ↓ F/B ratio (*Bacteroidetes* predominance) is associated with leanness or high-fiber diets [52].
A/P Ratio	Indicator of mucosal dysbiotic stress in our analysis. We evaluated the proportion of *Actinobacteria* (primarily beneficial *Bifidobacterium* spp.) relative to *Proteobacteria* in each sample. *Proteobacteria* (e.g., *Enterobacteriaceae* family) should generally make up no more than ~5% of the gut microbiome in healthy adults [53]. ↑ *Proteobacteria* levels are a hallmark of dysbiosis and inflammation, as many *Proteobacteria* are opportunistic pathogens that produce endotoxins and harmful metabolites like indoles, phenols, TMA, and H_2_S [54]. ↓ A/P ratio (i.e., *Proteobacteria* overgrowth concurrent with *Bifidobacteria* depletion) is often observed after antibiotic therapies or in inflammatory bowel conditions [55].
SCFA-Producing Flora	Our analysis quantified the relative abundance of key beneficial SCFA-producing taxa, such as *Faecalibacterium prausnitzii*, *Roseburia* spp., *Eubacterium* spp. (*E. rectale*, *E. hallii*), *Ruminococcus* spp., *Coprococcus* spp., and *Butyrivibrio* spp., as an index of the gut’s fermentative and anti-inflammatory capacity. These commensals ferment fiber into butyrate and propionate, which nourish colonic mucosa epithelial cells and alleviate local immune-inflammatory responses. Depletion of prominent butyrate producers (e.g., *F. prausnitzii*) has been linked to ↓ mucosal butyrate and correlates with ↑ intestinal inflammation [56].
Uremic Toxin-Producing Flora	Our analysis evaluated the microbial potential for generating uremic toxins—deleterious metabolites derived from bacterial amino acid fermentation. Key compounds of interest were indolic/phenolic metabolites and TMA. Indole, produced by bacterial tryptophan metabolism, is converted to indoxyl sulfate in the host, while bacterial fermentation of tyrosine/phenylalanine yields phenols (e.g., p-cresol). TMA is generated by certain gut bacteria from choline, betaine, or l-carnitine and is a precursor to TMAO, which influences cardiovascular risk [57]. The lab reports specifically flagged overgrowth of phenol-producing, indole-producing, or TMA-producing bacteria, when present (often *Proteobacteria* and certain *Clostridia*). Stool ammonia-producing bacterium levels were also measured, as another microbial byproduct of protein breakdown that can irritate the gut mucosa. These metrics allowed us to identify patients with a ↑ proteolytic fermentative burden, which is relevant because ↑ indoxyl sulfate and p-cresol have pro-oxidative, pro-inflammatory effects (linked to cardiovascular/renal risks) [58]. These parameters reflect inferred functional potential based on producer taxa and do not represent direct stool or serum metabolite quantification.
Mucosal Barrier Markers	Our analysis quantified *Akkermansia muciniphila* relative abundance (by specific 16S reads) and β-glucuronidase-bearing bacterial abundance (inferred from taxonomic profiling), as indicators of gut mucosal barrier function. *A. muciniphila* is a beneficial mucin-degrading bacterium that stimulates mucin production by goblet cells, thereby strengthening the intestinal lining [59]. ↓ *Akkermansia* abundance suggests a thinning of the protective mucin layer and has been associated with metabolic syndrome and inflammation. Stool β-glucuronidase, an enzyme produced by certain bacteria, deconjugates bilirubin and other glucuronides. ↑ β-glucuronidase activity can reflect dysbiosis where bacterial deconjugation of xenobiotics and intestinal mucus is increased, potentially leading to mucosal irritation and impaired clearance of toxins [60]. Healthy profiles should show adequate *Akkermansia* and normal-range β-glucuronidase-bearing bacterial levels.
Opportunistic Bacteria & Pathogens	All stool samples were assessed (16S sequencing) for any pathogenic, immunogenic or overabundant opportunistic bacteria: *Haemophilus*, *Acinetobacter*, *Proteus*, *Klebsiella*, *Enterobacter*, *Citrobacter*, *Serratia*, *Hafnia*, *Pseudomonas*, *Morganella*, *Providencia*, or atypical proliferation of *Escherichia coli* (especially toxin-producing strains)*, Enterococcus*, and *Lactobacillus*. The Biovis panel flags and reports on this specific list of (potentially) pathogenic genera. Mycological analysis was also performed: samples were cultured and checked by PCR for the presence of *Candida* yeasts: *C. albicans*, *C. glabrata*, *C. krusei*, *C. parapsilosis*, *C. tropicalis*, *C. dubliniensis*, *C. lusitaniae*. Parasitological examination (by multiplex PCR) for pathobionts and common pathogenic protozoan parasites (*Blastocystis hominis*, *Dientamoeba fragilis*, *Giardia lamblia*, *Entamoeba histolytica*, *Cryptosporidium* spp., *Cyclospora cayetanensis*) was also conducted. These assessments of exogenous pathogens ensured that observed microbiome changes were due to dysbiosis from CDI and its treatment, rather than new infections.
Methanogens & Sulfate-Reducers	Our analysis also quantified the relative abundance of methanogenic archaea (*Methanobrevibacter smithii*) and sulfate-reducing bacteria (*Desulfovibrio piger*, *Desulfomonas pigra*, *Bilophila wadsworthii*). Excessive methane production can slow intestinal transit and contribute to constipation and bloating (as seen in IBS-C) [61]. Sulfate-reducers generate H_2_S (by reducing dietary sulfate), a toxic gas that can damage the intestinal epithelium and which has been implicated in the pathogenesis of ulcerative colitis and other gut disorders [62]. ↑ H_2_S-producers or methanogens may explain patient symptoms (gas, altered motility) and guide dietary recommendations (e.g., ↓ sulfate or FODMAP intake).

Abbreviations: 16S = 16S ribosomal RNA; A/P = Actinobacteria-to-Proteobacteria ratio; CDI = Clostridioides difficile infection; F/B = Firmicutes-to-Bacteroidetes ratio; FODMAP = Fermentable Oligosaccharides, Disaccharides, Monosaccharides, and Polyols; H_2_S = hydrogen sulfide; IBS = Irritable Bowel Syndrome; IBS-C = Irritable Bowel Syndrome with Constipation; PCR = Polymerase Chain Reaction; SCFA = short-chain fatty acid; TMA = trimethylamine; TMAO = trimethylamine N-oxide. Symbols: ↑ = increased levels/above reference range; ↓ = decreased levels/below reference range.

## Data Availability

The commercial Biovis laboratory does not routinely release raw FASTQ files under the current service agreement; however, processed abundance tables and derived indices used for analysis are available from the corresponding author on reasonable request.

## Abbreviations

The following abbreviations are used in this manuscript:

16S rRNA	16S ribosomal RNA
A/P	Actinobacteria-to-Proteobacteria ratio
AAD	Antibiotic-associated diarrhea
CDI	Clostridioides difficile infection
CFU	Colony-forming unit
F/B	Firmicutes-to-Bacteroidetes ratio
FMT	Fecal microbiota transplantation
FODMAP	Fermentable oligosaccharides, disaccharides, monosaccharides, and polyols
H_2_S	Hydrogen sulfide
IBS	Irritable bowel syndrome
IBS-C	Irritable bowel syndrome with constipation
NGS	Next-generation sequencing
OB10	Omni-Biotic® 10
OTU	Operational taxonomic unit
PCA	Principal component analysis
PCR	Polymerase chain reaction
SCFA	Short-chain fatty acid
TMA	Trimethylamine
TMAO	Trimethylamine N-oxide
